# Supplementary material for: Ezrin enrichment on curved membranes requires a specific conformation or interaction with a curvature-sensitive partner
Source: eLife. 2018 Sep 20;7:e37262. doi: 10.7554/eLife.37262 (PMC6167055; doi:10.7554/eLife.37262)
Supplement: Transparent reporting form [file elife-37262-transrepform.docx]

***eLife’s* transparent reporting form**

We encourage authors to provide detailed information *within their submission* to facilitate the interpretation and replication of experiments. Authors can upload supporting documentation to indicate the use of appropriate reporting guidelines for health-related research (see [EQUATOR Network](http://www.equator-network.org/%20)), life science research (see the [BioSharing Information Resource](https://biosharing.org/)), or the [ARRIVE guidelines](http://www.plosbiology.org/article/info:doi/10.1371/journal.pbio.1000412) for reporting work involving animal research. Where applicable, authors should refer to any relevant reporting standards documents in this form.

If you have any questions, please consult our Journal Policies and/or contact us: [editorial@elifesciences.org](mailto:editorial@elifesciences.org).

**Sample-size estimation**

- You should state whether an appropriate sample size was computed when the study was being designed

*No computational estimation of sample size was performed.*

- You should state the statistical method of sample size computation and any required assumptions

*No statistical method was used to compute sample size.*

- If no explicit power analysis was used, you should describe how you decided what sample (replicate) size (number) to use

*At least 2- 3 replicates (named “sample preparations” in the article) were performed for almost all the experiments. For each replicates, if possible, more than 5 measurements were collected for data analysis.*

Please outline where this information can be found within the submission (e.g., sections or figure legends), or explain why this information doesn’t apply to your submission:

*Numbers of sample preparations (n) and sample numbers (N) were listed in figure legends when applicable.*

**Replicates**

- You should report how often each experiment was performed

*The number of replicate was indicated in each figure legend when applicable.*

- You should include a definition of biological versus technical replication

*Biological replicates were experiments performed independently at different days and technical replicates were samples collected in the same experiment.*

- The data obtained should be provided and sufficient information should be provided to indicate the number of independent biological and/or technical replicates

*Detailed information considering number of independent biological and technical replicates was indicated in the figure legends when applicable.*

- If you encountered any outliers, you should describe how these were handled

*Not Applicable*

- Criteria for exclusion/inclusion of data should be clearly stated

*All data collected were analyzed and presented.*

High-throughput sequence data should be uploaded before submission, with a private link for reviewers provided (these are available from both GEO and ArrayExpress)
*Not Applicable*

Please outline where this information can be found within the submission (e.g., sections or figure legends), or explain why this information doesn’t apply to your submission:

*In the figure legend for the following figures:*

*Figure 1, Figure 3, Figure 4, Figure 6, Figure 1–figure supplement 1, Figure 1–figure supplement 2, Figure 3–figure supplement 1, Figure 4–figure supplement 1, and Figure 6–figure supplement 1.*

**Statistical reporting**

- Statistical analysis methods should be described and justified
- Raw data should be presented in figures whenever informative to do so (typically when N per group is less than 10)
- For each experiment, you should identify the statistical tests used, exact values of N, definitions of center, methods of multiple test correction, and dispersion and precision measures (e.g., mean, median, SD, SEM, confidence intervals; and, for the major substantive results, a measure of effect size (e.g., Pearson's r, Cohen's d)
- Report exact p-values wherever possible alongside the summary statistics and 95% confidence intervals. These should be reported for all key questions and not only when the p-value is less than 0.05.

Please outline where this information can be found within the submission (e.g., sections or figure legends), or explain why this information doesn’t apply to your submission:

*- Data number and statistic method were listed in figure legends when applicable and in text.*

*- For many experiments, raw data was presented (Figure 1G, Figure 3D, Figure 3E, Figure 3F, Figure 3G, Figure 4, Figure 6C, Figure 6D, Figure 6G, Figure 1–figure supplement 1F, Figure 1–figure supplement 2B, Figure 1–figure supplement 2D, Figure 1–figure supplement 2E, Figure 3–figure supplement 1B, Figure 3–figure supplement 1C, Figure 6–figure supplement 1B and Figure 6–figure supplement 1E.*

*For data presented as averages and standard deviation, source files were provided.*

*- Statistical test, exact value of N, and precision measures (SD) were listed in the figure legends or main text when applicable.*

*- When applicable, p-value was reported.*

(For large datasets, or papers with a very large number of statistical tests, you may upload a single table file with tests, Ns, etc., with reference to sections in the manuscript.)

**Group allocation**

- Indicate how samples were allocated into experimental groups (in the case of clinical studies, please specify allocation to treatment method); if randomization was used, please also state if restricted randomization was applied
- Indicate if masking was used during group allocation, data collection and/or data analysis

Please outline where this information can be found within the submission (e.g., sections or figure legends), or explain why this information doesn’t apply to your submission:

*Not applicable for this study*

**Additional data files (“source data”)**

- We encourage you to upload relevant additional data files, such as numerical data that are represented as a graph in a figure, or as a summary table
- Where provided, these should be in the most useful format, and they can be uploaded as “Source data” files linked to a main figure or table
- Include model definition files including the full list of parameters used
- Include code used for data analysis (e.g., R, MatLab)
- Avoid stating that data files are “available upon request”

Please indicate the figures or tables for which source data files have been provided:

*- Figure 1–figure supplement 1C, 1D and 1G*

*- Figure 4–figure supplement 1A and 1B*
